# High-resolution maps of critical loads for sulfur and nitrogen in China

**DOI:** 10.1038/s41597-023-02178-z

**Published:** 2023-05-31

**Authors:** Xiaodong Ge, Qian Yu, Lei Duan, Yu Zhao, Maximilian Posch, Jiming Hao

**Affiliations:** 1 State Key Laboratory of Environmental Simulation and Pollution Control, School of Environment, Tsinghua University, Beijing, 100084 China; 2 State Key Laboratory of Pollution Control & Resource Reuse and School of the Environment, Nanjing University, Nanjing, Jiangsu 210023 China; 3 State Environmental Protection Key Laboratory of Sources and Control of Air Pollution Complex, Tsinghua University, Beijing, 100084 China; 4 International Institute for Applied System Analysis (IIASA), Schlossplatz 1, 2361 Laxenburg, Austria

**Keywords:** Environmental impact, Element cycles, Environmental chemistry

## Abstract

The critical load concept is an important scientific guideline for acid deposition control. It was not only a crucial scientific basis to determine the emission reduction targets in Europe, but also used in China’s air pollution control, especially the designation of two control zones. Currently, critical loads of sulfur and nitrogen are still exceeded in Europe, America, and East Asia (mainly in China), and need to be continuously updated to meet the demands of further emission reductions. Critical loads of China were calculated and mapped in the 2000s, but are not sufficiently accurate due to methodological and data limitations. Here we present the latest high-quality critical loads for China, based on high-resolution basic data on soil, vegetation, and atmospheric base cations deposition, and up-to-date knowledge on important parameters. Our data, which is going to be included in GAINS-China, can be used to assess the ecological benefits of nitrogen and sulfur reductions in China at a regional or national scale, and to develop mitigation strategies in the future.

## Background & Summary

Acid deposition, consisting (mainly) of nitrogen deposition and sulfur deposition, used to be one of the most serious environmental problems in Europe, North America, and East Asia since the 1960s^1,2^. It remains an important environmental problem in some developing countries such as India and Brazil, and is showing an increasing trend there^3^. Nitrogen and sulfur deposited into the environment may lead to acidification and eutrophication of terrestrial and aquatic ecosystems^4^. There have been many reports of acid deposition leading to fish kills and forest decline^5–7^. In order to effectively control the environmental impacts of acid deposition at minimal cost, the concept of critical loads was proposed, defined as the maximum amount of acid deposition that would not cause soil and surface water damage in the long term^8^. Critical loads are determined by the nature of the ecosystem (e.g., soil and vegetation), reflecting the tolerance of the ecosystem to acid deposition. Critical loads have been used in nitrogen and sulfur abatement around the world, even as the primary scientific guidance^9–11^. In Europe, they were used in the negotiations of the Second Sulfur Protocol and the “multi-pollutant, multi-effect” Protocol, as the scientific basis for emission reduction targets^12^. The US have also taken critical loads seriously, establishing the Critical Loads of Atmospheric Deposition Science Committee (CLAD) to promote the development, collaboration, and data sharing of critical loads^13^. Several federal agencies, such as the National Park Service (https://www.nps.gov/subjects/air/critical-loads.htm), have applied critical loads to ecosystem conservation practices. Currently, acid deposition is gradually declining in Europe and the US through the reduction of SO_2_ and NO_X_, but the critical loads are still exceeded in border areas there (e.g., exceedances of critical loads for eutrophication occurred on 58% of ecosystem area in Europe in 2020^14^; total area of any critical load exceedance in the US in 2025 is predicted to be 4.8 million km^2^)^15^. Therefore, Europe (www.icpmapping.org) and the US (http://nadp.slh.wisc.edu) are continuously updating critical loads to assess the benefits of further emission reduction.

Since the late 1970s, acid rain has gradually become one of the most concerning environmental issues in East Asia. And southern China is a hot spot for acid rain in East Asia^1^. The area affected by acid rain in China once exceeded 30% of the national land area^16^, and the highest wet deposition of sulfur in China was significantly higher than that in Europe and North America^1,17,18^. In order to scientifically evaluate the status of acid deposition and to guide emission reductions, Duan *et al*. first mapped the critical loads in China^9^, which were then used in the designation of two control zones (Acid Rain Control Zone and Sulfur Dioxide Pollution Control Zone)^19–21^. In recent years, China has made great efforts to reduce nitrogen and sulfur emissions, mainly for fine particulate matter (PM_2.5_) control, and acid deposition seems to have been greatly alleviated as a co-benefit^16^. However, based on Duan *et al*.’s results, Zhao *et al*. found that the critical load exceedance of sulfur remained at 2.5 Mt in 2015, and that of nitrogen was 1.1 Mt^22^. Furthermore, the particulate matter abatement reduces the tolerance of ecosystems to acid deposition, and reduction of ammonia also brings uncertainty in acid deposition control^23^. Therefore, the available critical load data in China (by Duan *et al*.^9^) cannot meet the need for an accurate assessment of acid deposition impacts currently in China. More importantly, Duan *et al*.’s critical loads were mapped more than two decades ago, thus the accuracy and resolution were limited by basic data and methods. For instance, they calculated denitrification and soil weathering rates based on soil type without localized parameters; vegetation uptake was roughly determined according to vegetation type. Generally, current critical load data has low spatial resolution and accuracy, and can no longer describe the latest situation of ecosystems due to changes in deposition, vegetation, and other environmental factors.

**Fig. 1:**
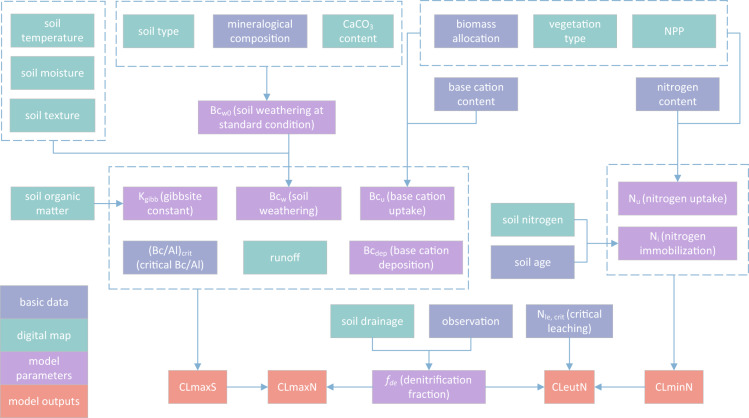
Methodology of mapping critical loads in China. Bc is the base cation (K + Ca + Mg); *K*_gibb_ is the gibbsite constant, which describes the balance between H^+^ and Al^3+^; (Bc/Al)_crit_ represents the critical molar ratio of Bc to Al in soil water; N_le, crit_ is the critical nitrogen leaching in runoff; *f*_de_ is the denitrification fraction of the net input nitrogen. Further details can be found in “Methods”.

To meet the demand for assessment of acid deposition status in China, we developed a high-resolution (1 km × 1 km) critical load dataset for soils, based on the Steady-state Mass Balance (SMB) model^24,25^, including the maximum critical load of sulfur (CL_max_ (S)), the minimum (CL_min_ (N)) and maximum (CL_max_ (N)) critical load of nitrogen, and the critical load of nutrient nitrogen (CL_nut_ (N)). Our results used high-resolution geographic, vegetation, and meteorological data, and up-to-date knowledge from literature, to update and refine the key parameters. The data calculation methodology is shown in Fig. 1. Our datasets can be used for the evaluation of the ecological impacts of acid deposition from regional to national scales in China.

## Methods

Our calculation method refers to the European manual on modelling and mapping critical loads, which are based on the principles of conservation of mass and charge^26^. It has to be noted that the SMB model is a steady-state model, i.e. all inputs and outputs considered are stable over time^24^, so finite reservoirs such as ion exchange are not included.

**Fig. 2:**
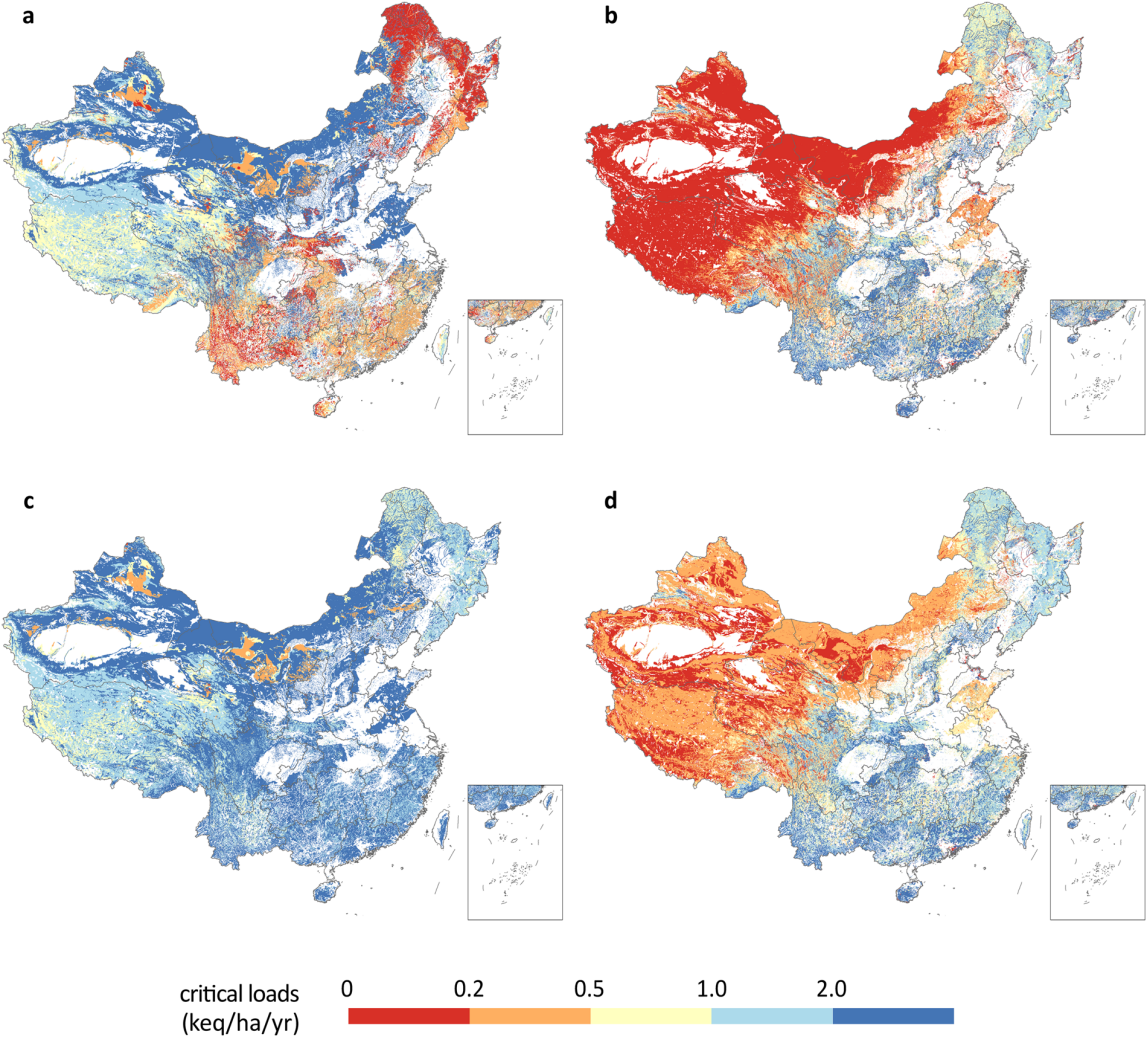
Critical loads of sulfur and nitrogen in China. The white colour in the map represents croplands or area with no vegetation represents croplands or area with no vegetation, where critical loads cannot be defined. (**a**) maximum critical load of sulfur; (**b**) minimum critical load of nitrogen; (**c**) maximum critical load of nitrogen; (**d**) critical load of nutrient nitrogen.

The maximum critical load of sulfur, CL_max_ (S) (Fig. 2a), was calculated as

1 $${{\rm{CL}}}_{{\rm{\max }}}\left({\rm{S}}\right)={{\rm{Bc}}}_{{\rm{dep}}}+{{\rm{Bc}}}_{{\rm{w}}}-{{\rm{Bc}}}_{{\rm{u}}}-{{\rm{ANC}}}_{{\rm{le}},{\rm{crit}}}$$

where Bc is the sum of base cation (i.e., K + Mg+Ca); the subscript dep stands for deposition, w stands for soil weathering, u stands for net uptake by plants, and le stands for leaching; Na is not included in Bc, because plants do not take up Na; ANC_le, crit_ is the acceptable limit of the leaching of acid neutralising capacity (ANC), which is given by:

2 $${{\rm{ANC}}}_{{\rm{le}}}=-Q\times \left(\left[{\rm{H}}\right]+\left[{\rm{Al}}\right]\right)$$

where *Q* is the runoff, [H] ([Al]) is the equivalent concentration of H^+^ (Al^3+^) in the runoff. Further calculation will be introduced in “Critical chemical criteria”.

Nitrogen undergoes a more complex biogeochemical cycle than sulfur. As long as the nitrogen deposition is not too high, it is assumed that the deposited nitrogen is all taken up by vegetation or immobilized and therefore has no environmental impact. Therefore, the minimum critical load of nitrogen, CL_min_ (N) (Fig. 2b), is defined as

3 $${{\rm{CL}}}_{{\rm{\min }}}\left({\rm{N}}\right)={{\rm{N}}}_{{\rm{u}}}+{{\rm{N}}}_{{\rm{i}}}$$

where N_u_ and N_i_ are the net nitrogen taken up by plants and long-term nitrogen immobilization, respectively.

When nitrogen deposition exceeds CL_min_ (N), some of the excess nitrogen would be denitrified, and the rest would leach and cause acidification. Therefore, the maximum critical load of nitrogen, CL_max_ (N) (Fig. 2c), is defined as

4 $${{\rm{CL}}}_{{\rm{\max }}}\left({\rm{N}}\right)={{\rm{CL}}}_{{\rm{\min }}}\left({\rm{N}}\right)+\frac{{{\rm{CL}}}_{{\rm{\max }}}\left({\rm{S}}\right)}{1-{f}_{{\rm{de}}}}$$

where *f*_de_ is the denitrification fraction.

Excess nitrogen may also lead to eutrophication. From this perspective, we can define the critical load of nutrient nitrogen, CL_nut_ (N) (Fig. 2d) as:

5 $${{\rm{CL}}}_{{\rm{nut}}}\left({\rm{N}}\right)={{\rm{CL}}}_{{\rm{\min }}}\left({\rm{N}}\right)+\frac{{{\rm{N}}}_{{\rm{le}},{\rm{crit}}}}{1-{f}_{{\rm{de}}}}$$

where N_le, crit_ is the acceptable limit to the leaching of nitrogen.

The parameters of the available critical loads were mainly determined by soil and vegetation types, while the characteristics of soil and vegetation (e.g., soil texture, soil moisture, and vegetation productivity) vary widely in space and are related to many factors. Therefore, the accuracy needs to be improved urgently. Instead, we used the latest high-resolution digital maps (Fig. 1), and combined up-to-date knowledge to determined important parameters such as soil weathering, denitrification, and nitrogen immobilisation, which greatly improved the accuracy and resolution of the critical loads. In the following we will describe in detail the determination of the key parameters.

### Soil weathering

**Fig. 3:**
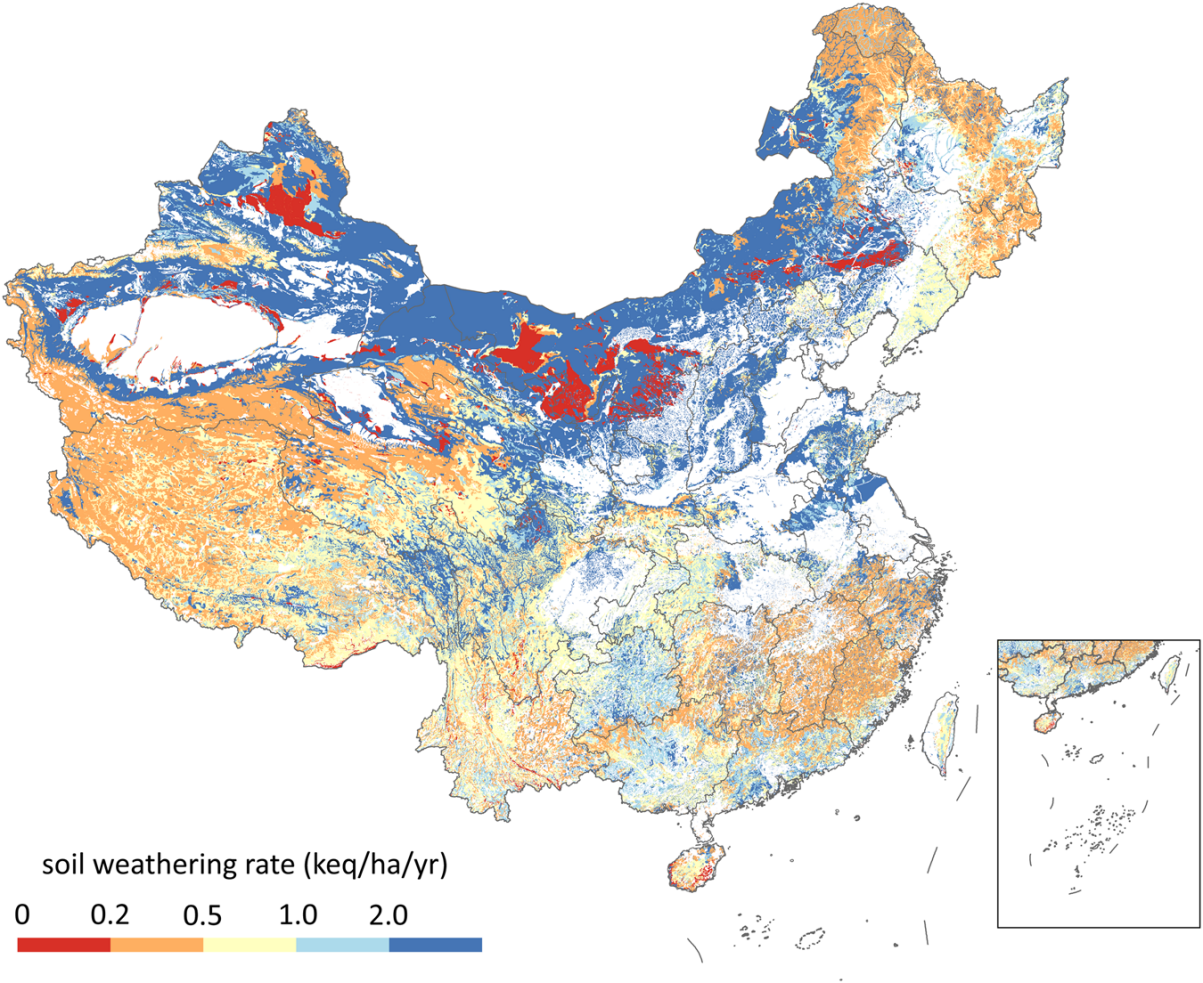
Soil weathering rate in China (corrected with soil moisture, SSA, and soil temperature).

Soil weathering rate is determined based on the mineralogical composition, the physical properties (e.g., texture), and environmental factors (e.g., temperature) (Fig. 3). First, we used the PROFILE model^27^ to calculate the baseline weathering rate at standard conditions (temperature = 8 °C, density = 1.2 g/cm^3^, specific surface area = 1.1 × 10^6^ m^2^/m^3^) for each soil type. Mineralogical composition data for typical soil types was compiled by Duan^9^. The 1 km × 1 km map of soil types in China was taken from Resource and Environment Science and Data Center (https://www.resdc.cn). Some soils in China contain calcium carbonate (CaCO_3_) especially in arid areas, but that was excluded in the calculation when the content was lower than 0.5% since it might be depleted during the long-term acid deposition. The weathering rate was calculated as CaCO_3_ content (%) times 0.82 keq/ha/yr when the CaCO_3_ content was higher than 0.5%. The soil CaCO_3_ content was from the Harmonized World Soil Database (HWSD)^28^.

The standard conditions are the default conditions in PROFILE and are designed to calculate weathering rates considering only the differences in the mineralogical composition. The weathering rates were then corrected with soil moisture, specific surface area (SSA), and soil temperature:

6 $${{\rm{Bc}}}_{{\rm{w}}1}={C}_{{\rm{sw}}}\times \frac{{\rm{SSA}}}{1.1\times 1.2}\times \exp \left(\frac{A}{T}-\frac{A}{281}\right)\times {{\rm{Bc}}}_{{\rm{w}}0}$$

where Bc_w0_ is the baseline weathering rate, *C*_sw_ is a correction factor for soil moisture, *A* is a constant (3600 K as recommended) for temperature correction, and *T* is the soil temperature. Soil temperature data was from the National Tibetan Plateau Data Center^29,30^. *C*_sw_ ranges from 0.7 (soil moisture < wilting moisture) to 1.3 (soil moisture > water holding capacity) and linear interpolation were used to determine it in areas of moderate moisture. The soil moisture data was from the National Earth System Science Data Center^31^. The numbers 1.1, 1.2, and 281 in Eq. (5) means the SSA of 1.1 × 10^6^ m^2^/m^3^, bulk density of 1.2 × 10^3^ kg/m^3^, and soil temperature of 281 K, which are the values at standard conditions.

SSA in Eq. (5) was estimated with

7 $${\rm{SSA}}=\left(0.3{X}_{{\rm{sand}}}+2.2{X}_{{\rm{silt}}}+8.0{f}_{{\rm{clay}}}{X}_{{\rm{clay}}}\right)\times \left(1-S\right)\times {\rho }_{{\rm{soil}}}$$

where *X*_sand_, *X*_silt_, *X*_clay_, and *S* mean the fraction of sand, silt, clay, and gravel in the soil; *ρ*_soil_ is the soil bulk density; *f*_clay_ is a correction factor^32^:

8 $${f}_{{\rm{clay}}}=1-\frac{{X}_{{\rm{clay}}}^{2.5}}{{X}_{{\rm{clay}}}^{2.5}+0.35}$$

Soil bulk density and texture (sand, silt, clay, and gravel content) data were from HWSD^28^.

### Net growth uptake by plants

**Fig. 4:**
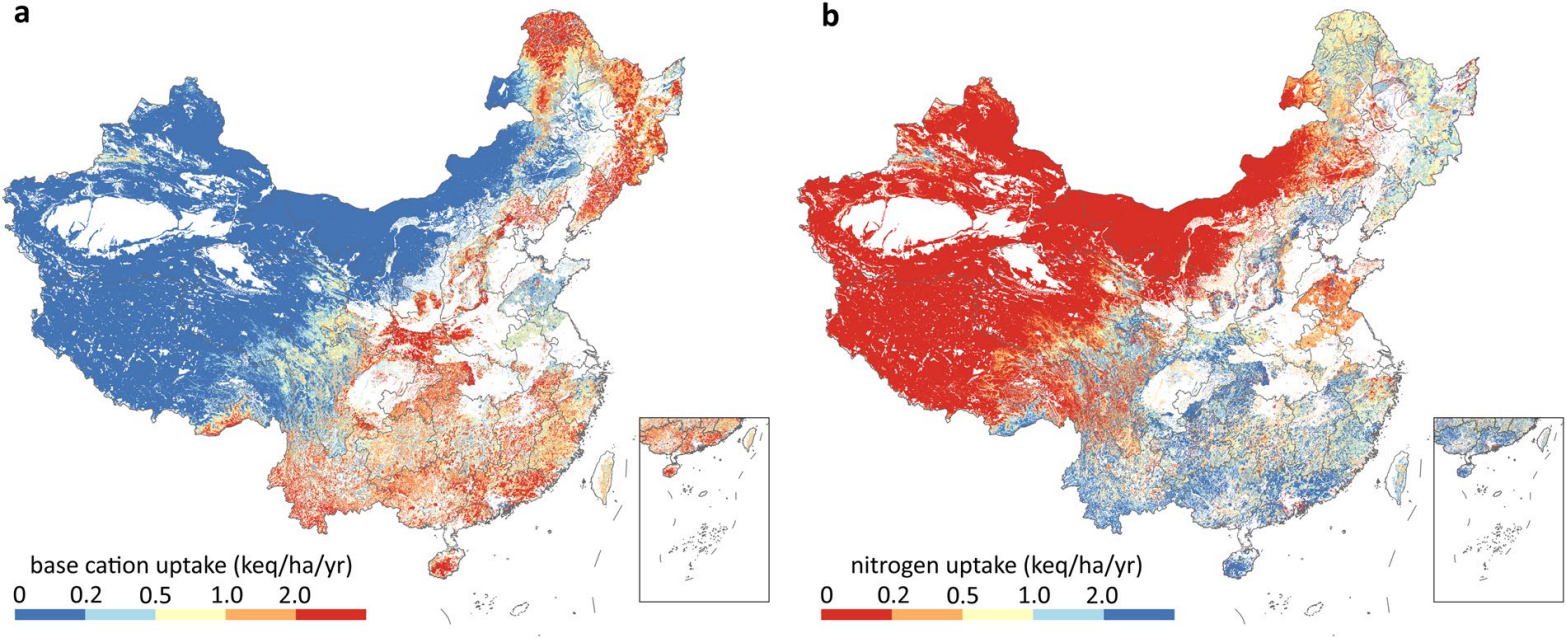
Net growth uptake of base cation and nitrogen by vegetation in China. The white colour in the map means no vegetation or croplands, where the net uptake is zero. (**a**), uptake of base cation; (**b**), uptake of nitrogen. Notice that the two figures use opposite colour schemes to indicate the risk of acid deposition.

Net uptake by plants means the net removal of nitrogen and base cations from the ecosystem (Fig. 4). The nitrogen contained in the trunks of trees, branches of shrubs, and the above-ground parts of grasslands was treated as nitrogen removed from the ecosystem, assuming scientific forest harvesting and grazing management were adopted. The net uptake of nitrogen or base cations was computed as:

9 $${{\rm{N}}}_{{\rm{u}}}\left({\rm{or}}\,{{\rm{Bc}}}_{{\rm{u}}}\right)={\rm{NPP}}\times {p}_{i}\times {C}_{{\rm{N}}}\left({\rm{or}}\,{C}_{{\rm{Bc}}}\right)$$

where NPP refers to net primary productivity, *p*_*i*_ is the proportion of biomass in the considered plant part (e.g., trunks of trees), and *C*_N_ (*C*_Bc_) is the nitrogen (base cation) content of plants. The annual NPP data modelled by Global Production Efficiency Model from 2000 to 2010 was from Resource and Environment Science and Data Center and was averaged to represent long-term NPP. Biomass proportion and elemental content data were collected by Duan *et al*.^33^ and linked to the vegetation map of China, which is from National Cryosphere Desert Data Center^34^.

### Nitrogen immobilization

**Fig. 5:**
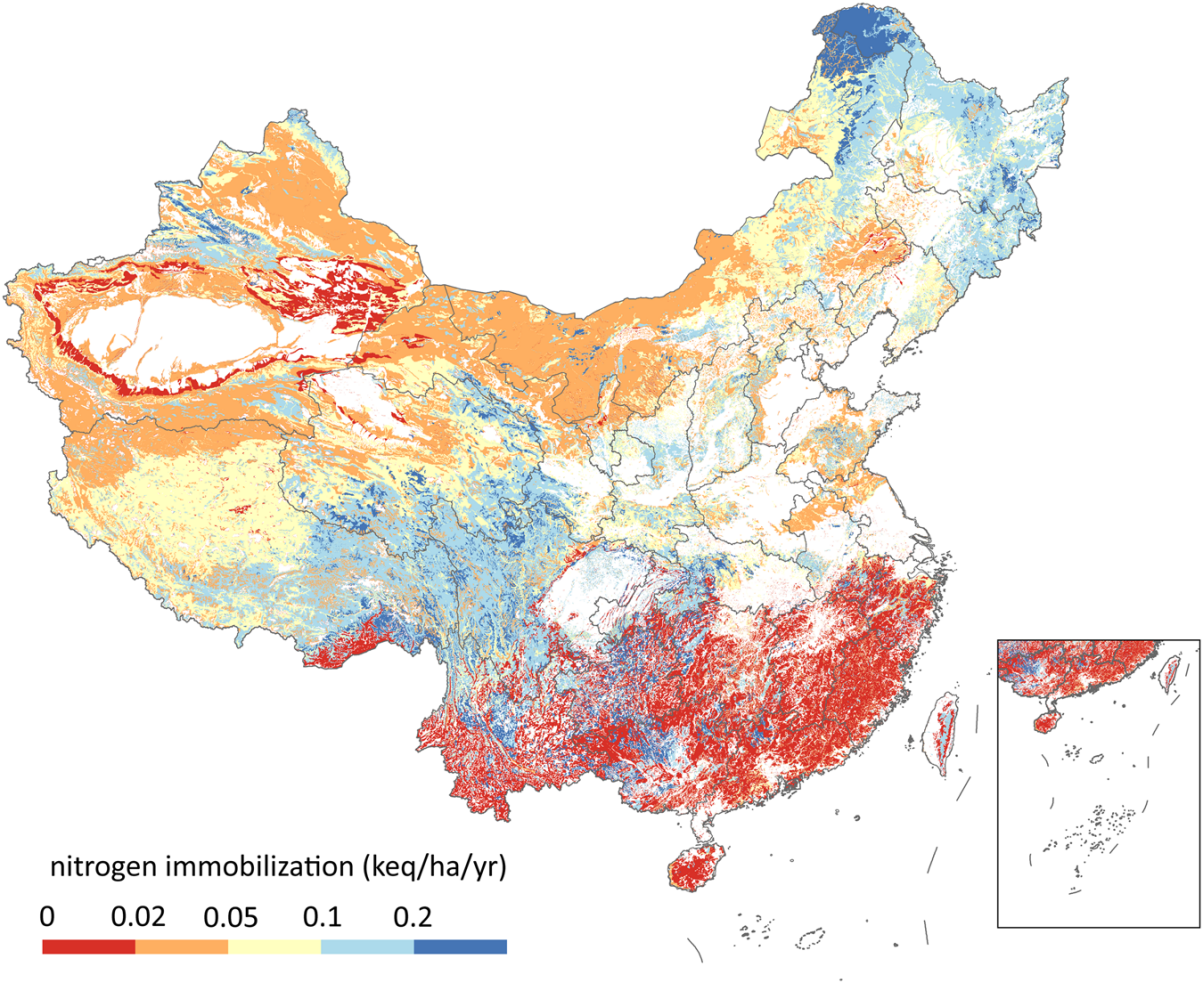
Nitrogen immobilization in the soils in China.

Nitrogen immobilization refers to the conversion of inorganic nitrogen to stable organic nitrogen in the soil (Fig. 5). The long-term net nitrogen immobilization was estimated by the soil nitrogen content divided by soil age^26^. The soils were divided into three categories, Skeletol Primitive Soils, Ferralisols, and others, whose ages were set to 1500 years, 130000 years, and 5000 years, respectively, according to measurements and soil type^35^. Soil nitrogen content was from National Tibetan Plateau Data Center^36,37^.

### Denitrification

We used the denitrification fraction *f*_de_ based on the observation data on denitrification for main forest types in China^38,39^ (Table 1). *f*_de_ in the other areas was determined according to the soil drainage status, which ranged from 0 for excessively drained soils to 0.8 for very poorly drained soils. Soil drainage data was obtained from the HWSD^28^. In order to prevent overestimating *f*_de_ of coarse soils, we set *f*_de_ = 0.1 when SSA<2 × 10^6^ m^2^/m^3^. The results were consistent with observations^38,39^.

**Table 1:** Denitrification fraction (*f*_de_) of main forest types in China^38,39^.

forest type	tropical broadleaf	temperate coniferous	temperate broadleaf	subtropical
***f***_**de**_	0.65	0.20	0.25	0.35

### Base cation deposition

**Fig. 6:**
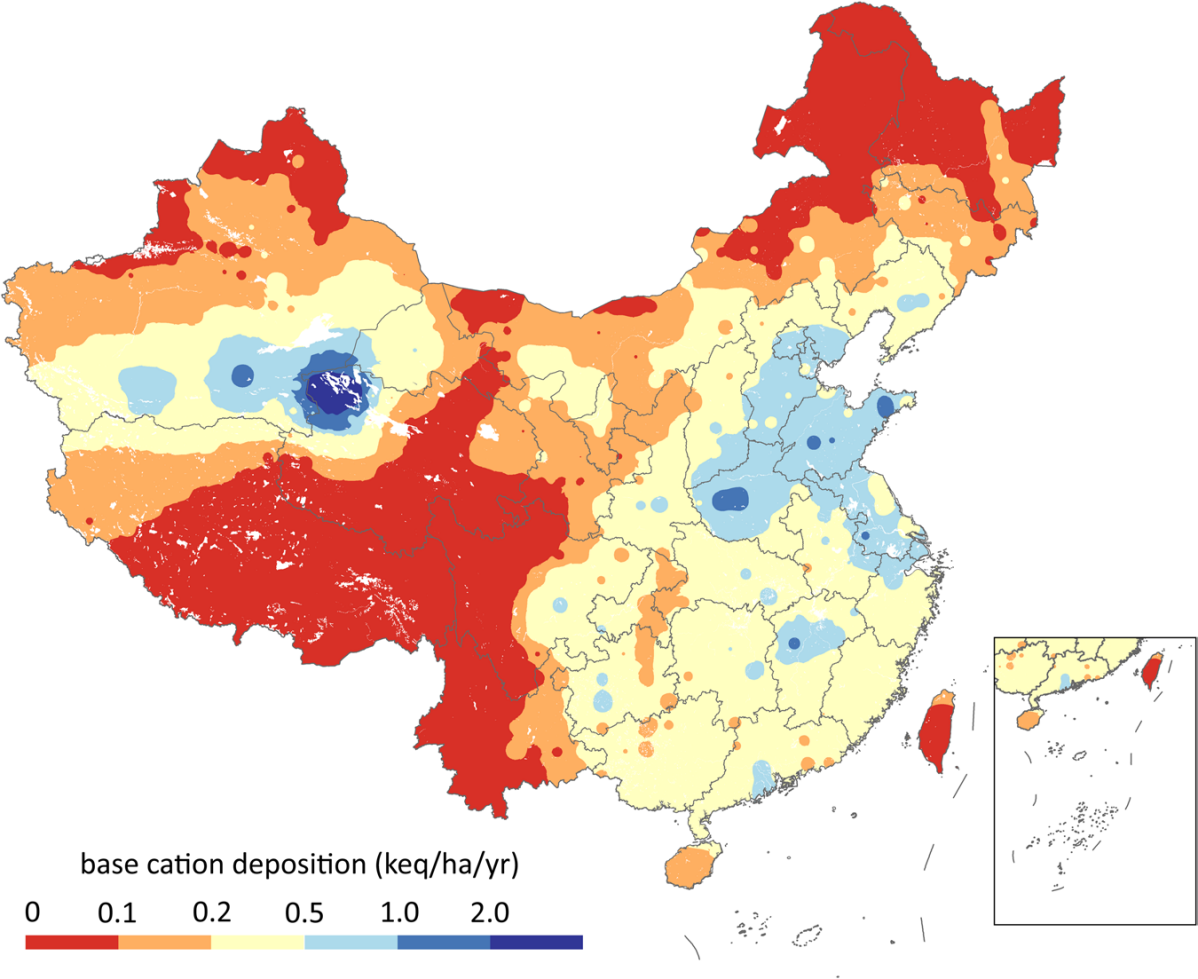
Base cation deposition in China in 2015.

Base cation deposition (Fig. 6) was simulated using a multi-layer dynamic Eulerian model developed by Duan *et al*.^40^ The model inputs include Bc emission inventory and meteorological data. The precipitation dataset was derived from the Global Precipitation Climatology Project^41^, and other meteorological data was stem from European Center for Medium-Range Weather Forecasts^42^. Bc emission inventory was calculated as:

10 $${{\rm{Bc}}}_{k}=\sum _{i.j}{{\rm{PM}}}_{i,j}\times {\omega }_{k,i,j}$$

where PM is the particulate matter emissions, *ω* is the mass fraction of Bc in particulate matter, and *i, j* and *k* represent province, sector, and Bc species, respectively. PM emission referred to Xia *et al*.^43^, and fractions of Bc species were from our previous research^44^.

### Critical chemical criteria

To protect plants from the damage of aluminium, we set a limit to the base cation to aluminium ratio (Bc/Al) for each ecosystem type. Critical ANC_le_ in Eq. (2) is then given by:

11 $${{\rm{ANC}}}_{{\rm{le}},{\rm{crit}}}=-{Q}^{\frac{2}{3}}\times {\left(1.5\times \frac{{{\rm{Bc}}}_{{\rm{dep}}}+{{\rm{Bc}}}_{{\rm{w}}}-{{\rm{Bc}}}_{{\rm{u}}}}{{K}_{{\rm{gibb}}}\times {\left(\frac{{\rm{Bc}}}{{\rm{Al}}}\right)}_{{\rm{crit}}}}\right)}^{\frac{1}{3}}-1.5\times \frac{{{\rm{Bc}}}_{{\rm{dep}}}+{{\rm{Bc}}}_{{\rm{w}}}-{{\rm{Bc}}}_{{\rm{u}}}}{{\left(\frac{{\rm{Bc}}}{{\rm{Al}}}\right)}_{{\rm{crit}}}}$$

where *K*_gibb_ is the gibbsite equilibrium constant, describing the balance between H^+^ and Al^3+^; (Bc/Al)_crit_ is the molar critical base cation to aluminium ratio. *K*_gibb_ was determined according to the soil organic matter content (Table 2). Soil organic matter content was obtained from National Tibetan Plateau Data Center^36,37^; The 1 km × 1 km runoff data of China was converted from zonal runoff map^45^; (Bc/Al)_crit_ for each ecosystem type was taken from Duan^9^ and the manual^26^.

**Table 2:** Relationship between gibbsite equilibrium constant (*K*_gibb_) and soil organic matter content.

**Soil organic matter (%)**	<1.5	1.5–3.5	3.5–5.0	5.0–15	15–30	>30
***K***_**gibb**_ **(m**^**6**^**/eq**^**2**^**)**	9500	3000	950	300	100	9.5

N_le, crit_ in Eq. (4) means the critical leaching of nitrogen, which is defined to protect the ecosystem from eutrophication. N_le, crit_ for each ecosystem type was taken from Duan^9^ and the manual^26^.

## Data Records

The data are freely available at National Tibetan Plateau Data Center^46^ and is going to be included in Greenhouse Gas - Air Pollution Interactions and Synergies model (https://gains.iiasa.ac.at/models/). The dataset consists of four TIF files and a ‘readme’ file. The TIF files give the critical loads (CL_max_ (S), CL_min_ (N), CL_max_ (N), and CL_nut_ (N)) of China. The ‘readme’ file explains the units and additional information for critical loads.

## Technical Validation

The SMB model has been extensively used around the world, and we calibrated the model parameters with reference to the latest research. However, critical loads are long-term attributes of ecosystems, which is difficult to validate by experiments. Therefore, quality control of the input data is the main approach to ensure the reliability of output at present.

The high-resolution maps we used (e.g., soil texture, soil organic matter) are peer-reviewed data or data from authoritative data repositories (e.g., soil type, vegetation type), which have high quality. For some other data (e.g., physiological data of plants, critical chemical criteria), we obtained them from literature or manuals, which can also be considered reliable.

Although *f*_de_ is very complex and related to many factors besides forest type, there is no widely accepted model to calculate *f*_de_ yet. We summarized the observations on *f*_de_ from literature for different forest type, which can better represent the actual situation in China compared to commonly used empirical relationships.

Soil ages were determined by soil type base on literature. Since the contribution of nitrogen immobilisation to critical loads is negligible, our rough estimation of soil ages would not cause large errors. In previous research^9^, a uniform soil age was used for all soil types because the available data for soil ages was very limited.

The main uncertainty arises from the estimation of soil weathering rates, as the mineralogical composition of soils is poorly studied and fragmented. However, weathering rates of the same soil type are relatively close due to the similar soil formation process and therefore the method we currently used is acceptable^47^. Generally, this study provides high quality estimation of critical loads in China under existing conditions.

## Data Availability

All calculations were done in ESRI ArcGIS 10.5 and no other computer code was used.
